# Determination of the Influence of Hydraulic Additives on the Foaming Process and Stability of the Produced Geopolymer Foams

**DOI:** 10.3390/ma14175090

**Published:** 2021-09-06

**Authors:** Michał Łach, Kinga Pławecka, Agnieszka Bąk, Katarzyna Lichocka, Kinga Korniejenko, An Cheng, Wei-Ting Lin

**Affiliations:** 1 Chair of Materials Engineering, Faculty of Material Engineering and Physics, Cracow University of Technology, Jana Pawła II 37, 31-864 Cracow, Poland; michal.lach@pk.edu.pl (M.Ł.); agnieszka.bak@pk.edu.pl (A.B.); katarzynalichocka9@gmail.com (K.L.); kkorniejenko@pk.edu.pl (K.K.); 2 Department of Civil Engineering, National Ilan University, No. 1, Sec. 1, Shennong Rd., Yilan City 260, Taiwan; ancheng@niu.edu.tw (A.C.); wtlin@niu.edu.tw (W.-T.L.)

**Keywords:** geopolymer, foaming process, sustainable material technologies, surfactant, hybrid additives, thermal conductivity

## Abstract

The research described in this article was aimed at determining the influence of hydraulic additives on the foaming process and the stability of the produced geopolymer foams. These foams can be used as insulation materials to replace the currently commonly used insulations such as expanded polystyrene or polyurethane foams. Geopolymers have low thermal conductivity, excellent fire- and heat-resistant properties, and have fairly good mechanical properties. Research on foamed materials shows that they have the highest class of fire resistance; therefore, they are most often used as insulation products in construction. Geopolymer foams were made of aluminosilicate materials (fly ash) and foaming agents (H_2_O_2_ and Al powder), and the stabilizers were gypsum and portland cement. Additionally, surfactants were also used. It was found that better foaming effects were obtained for H_2_O_2_—it is a better foaming agent for geopolymers than Al powder. When using a hydraulic additive—a stabilizer in the form of cement—lower densities and better insulation parameters were obtained than when using gypsum. Portland cement is a better stabilizer than gypsum (calcium sulfates), although the effect may change due to the addition of surfactants, for example.

## 1. Introduction

The use of lightweight concrete (<1850 kg/m^3^) in the construction industry has many advantages including better thermal insulation, reduced dimensions and dead loads, savings in steel reinforcement and lower transportation costs. Unfortunately the disadvantage of lightweight concrete is that it is less resistant to cracking and more brittle than standard concrete. This is due to the use of lightweight materials, which are weaker than the traditional cement matrix [1,2,3].

**Figure 1:**
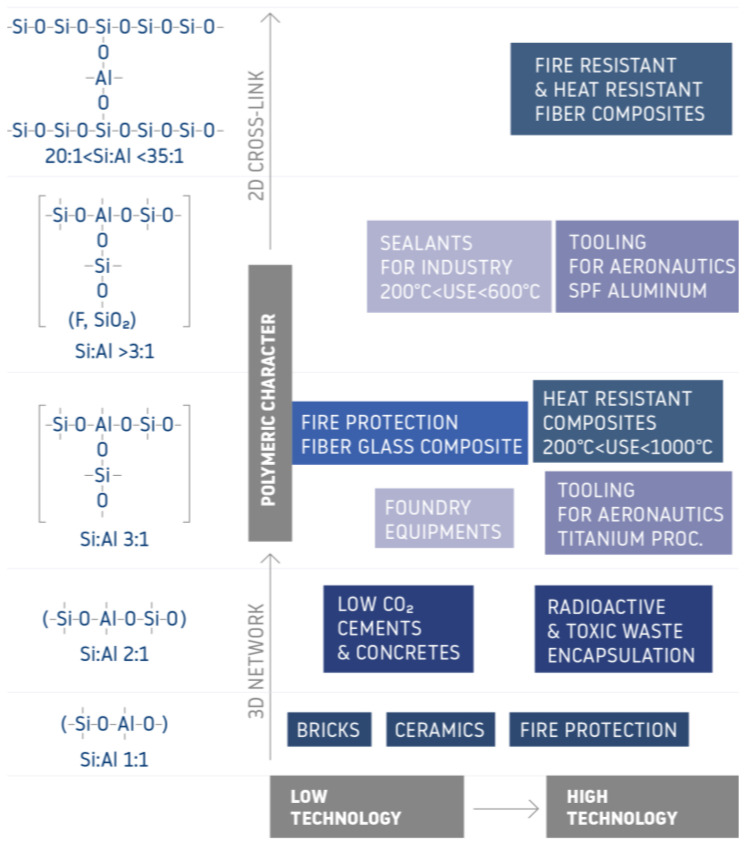
Possible applications of geopolymers depending on the structure (based on [15]).

Geopolymer foams are inorganic, porous materials whose development has been very dynamic in recent years. Geopolymer materials may be synthesized at elevated or ambient temperatures by alkaline activation of industrial waste products (fly ash, slag) or materials of geological origin (metakaolin, volcanic tuff) [4,5,6]. All over the world, access to starting materials (raw materials) is very common, and the demand for this type of materials also seems to be growing, hence their wide potential use and attractiveness. The production of geopolymers is very economical as well as safe for people and the environment. Due to the constant efforts to obtain the best insulation parameters of buildings and to achieve the so-called passivity, many laboratories around the world are working on solutions for new insulating materials with different properties from those commonly used. A properly selected thermal insulation material is a guarantee of effective and properly functioning thermal insulation of a building. In winter, proper wall insulation will allow you to reduce the heating costs of the building, while in summer, effective insulation will protect against intense heating. Geopolymers seems to be one of the most interesting alternatives to popular insulation materials here. Geopolymers have about 50% lower thermal conductivity value (<0.70 [W/(m × K)] compared to Portland cement materials. The thermal insulation properties can be further improved by introducing voids (pores) into the geopolymer matrix [7,8]. Geopolymer foam is a lightweight aluminosilicate structure (after alkaline activation) with a high degree of porosity. This material is also referred to as foam concrete, which by definition is a cellular concrete with a large number of hollow spaces, with or without the addition of aggregate [9]. They have good thermal insulation and acoustic properties [10,11,12,13] as well as mechanical properties, although their compressive strength is lower than that of solid (not foamed) geopolymers [10,14]. The comparison of fire-resistant properties of geopolymers and other materials is shown in Figure 1 [15]. Lyon et al. in their paper studied the behavior of composite materials in a fire test (ISO 9705). They described that geopolymer composites will never catch fire or produce smoke compared to other engineering thermoplastics [16].

The following materials can be used as foaming agents: aluminum powder, sodium hypochlorite, silica dust, and hydrogen peroxide. These agents react with alkaline activators to create pores in the geopolymer structure [10,12,15]. In the case of aluminum powder, voids are formed according to the following hydrogen release reaction [16,17,18,19,20]:

2Al + 2OH^−^ + 6H_2_O » 2[Al(OH)_4_]^−^ + 3H_2_

Hydrogen peroxide decomposes into water and oxygen. The release of gaseous oxygen causes the formation of empty air spaces [16,21,22]:

2 H_2_O_2_ » 2H_2_O + O_2_

The process of producing the foam itself is quite simple and well known. The problem arises when the porous structure produced by foaming agents is stable only for a short time and then falls after a while (before the bonding process—geopolymerization). Even if the pore formation efficiency is very high, the foams produced will have a high density and low insulating properties if the porous spatial structure is not stable. There are various ways to prevent the foams from falling. The surfactant additive is the most common.

For the stabilization of fresh geopolymer foam, nonionic surfactants are also added, e.g., Tween 80, and Polyoxyethylene 20 sorbitan monooleate—C_64_H_124_O_26_ (VWR BDH Prolabo) and Triton X-100, a polyethylene glycol tertoctylphenyl ether—C_14_H_22_O (C_2_H_4_O) n, n = −10 (Sigma-Aldrich) [23]. As a surfactant, for example, pork lard or butter [22] was used. Olive oil has also been used as a surfactant [24]. Agents such as (e.g., Sika Lightcrete 02) containing e.g., 40 wt% solutions of fatty acid, amide and sodium salt of C14-C16 sulphonic acid in water are also often added. Chemicals are also added to reduce the viscosity, e.g., polyacrylic acid (Dolapix CE-64) [25]. Good results are also obtained with calcium stearate [26].

This paper presents the results of research on foamed geopolymer composites with the addition (apart from surfactants) of hydraulic binders stabilizers such as Portland cement and gypsum. The research results show that, in general, adding cement improves all properties of fly ash-based geopolymers, except workability [27]. There are also studies of geopolymer–cement hybrids that confirm good strength properties and other such solutions [28]. The authors focused on determining the effect of adding hydraulic binders hydraulic binders (Portland cement and building gypsum) combined with surfactants (surfactants) on the foaming process and stability of geopolymer foams based on fly ash and construction building sand. The main assumption of the research was to obtain a material with the lowest possible thermal conductivity coefficient (lower or equal to the Styrofoam) while maintaining low specific gravity of the finished product. Geopolymer materials are durable and nonflammable, so they could compete with traditional insulation materials used in the construction industry. The aim of the research was to determine how the hydraulic binders used as stabilizers of the produced geopolymer foams will behave in combination with surfactants. The main novelty of the presented results is that the authors have carried out the analysis of the influence of both hydraulic additives and surfactants on the properties of the produced geopolymer foams. Additives such as cement or gypsum were aimed at foam stabilization and its faster setting while surfactants were aimed at changing the surface tension. So far, no such analyses have been presented in articles (published studies), although the effect of both types of these additives is commonly known.

## 2. Materials and Methods

Geopolymer foams were made based on fly ash, which came from the Skawina Heat and Power Plant (Skawina, Poland). Table 1 shows the oxide composition of the ash determined using the XRF method.

Technical sodium hydroxide in the form of flakes and an aqueous solution of sodium silicate R-145 with a molar module of 2.5 and a density of about 1.45 g/cm^3^ were used for the production of geopolymers. The added make-up water was “mains” water; no distilled water was used. The alkaline solution was prepared in such a way that the solid sodium hydroxide was poured over an aqueous solution of sodium silicate and water. The solution was thoroughly mixed and allowed to equilibrate until reaching a constant concentration and temperature. For each sample, the same amount of construction sand (100 g) and fly ash were used. Hydraulic additives used in the tests were building gypsum and CEM 52.5 Portland cement. Two types of agents were used as stabilizers: Syringaldehyde [29], in the form of a beige powder and Poly(ethylene glycol) diacrylate; and (Average Mn575) [30], in the form of a transparent liquid. Table 2 shows the characteristics of the stabilisers used to produce the foamed geopolymers analysed.

The solid components, i.e., fly ash, sand, and stabilizer (Syringaldehyde), were mixed dry until a homogeneous mixture was obtained, and then the alkali solution was added and mixed thoroughly. Average Mn575 (the other stabilizer) was added after obtaining a homogeneous mixture of fly ash and sand along with the alkali activator. Mixing was carried out in a laboratory mixer (LMB-s [31]) for about 15 min. After obtaining a homogeneous mass with a densely plastic consistency, foaming agents in the form of Al or H_2_O_2_ powder were added, then the mixtures were transferred to appropriate molds. After 24 h, the samples were removed and demolded.

**Figure 2:**
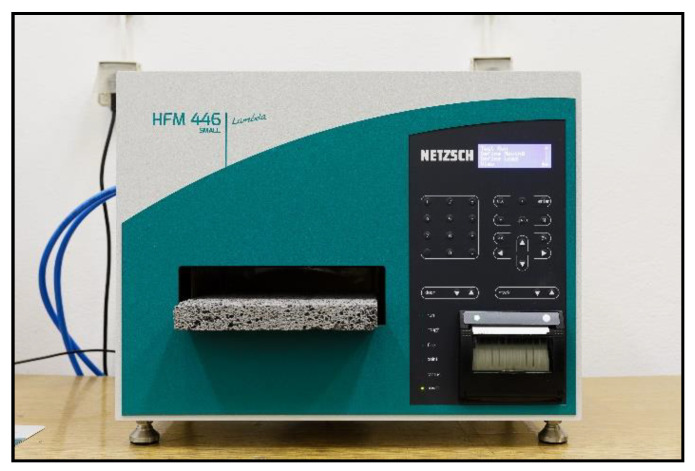
HFM 446 Lambda Series—Heat Flow Meter for Testing Insulation Materials.

The heat conduction coefficient was tested on the HFM 446 plate apparatus (Wittelsbacherstrasse, Germany). Figure 2 shows the thermal conductivity testing machine used in the above test.

In a heat flow meter (HFM), the test specimen is placed between two heated plates controlled to a user-defined mean sample temperature and temperature drop to measure heat flowing through the specimen. The sample thickness (L) corresponds to the actual sample dimension or to match the desired thickness of a compressible sample. The heat flow (Q) through the sample is measured by two calibrated heat flux transducers covering a large area of both sides of the specimen. After reaching a thermal equilibrium, the test is done. For the tests, panels with dimensions of approximately 200 mm × 200 mm × 25 mm were made, which were dried to a constant weight after 28 days of maturation and tested.

Density measurements were made using the geometric method. The actual density of the foamed geopolymers produced was determined using a geometric method. The density was determined as the average of measurements for 3 samples of a given type of material. Samples were measured with a laboratory caliper with the measurement accuracy of 0.01 mm, and the mass of samples was determined using a RADWAG PS 200/2000.R2 laboratory analytical balance (maximum load: 200/2000 g; reading accuracy: 0.001/0.01 g).

**Figure 3:**
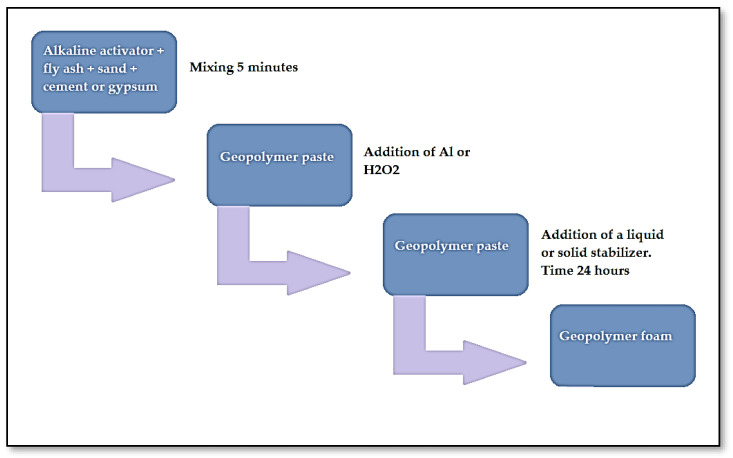
The process of producing foamed geopolymers with the addition of stabilizers.

Figure 3 below shows schematically the process of producing foamed geopolymers with the addition of various types of stabilizers.

## 3. Results

Table 3 and Table 4 below show the designation of the samples with an indication of the ingredients used in their production. All variants of geopolymeric foam composites with additives (stabilizers and surfactants) were presented. An alkaline solution of 10 M NaOH was used for all samples together with a water glass. Table 3 shows the determinations and composition of the samples foamed with 30 mL (in each case) of H_2_O_2_ (concentration 30%), while Table 4 shows the determinations and composition of the samples foamed with 1.5 g (in each case) of aluminum powder.

**Figure 4:**
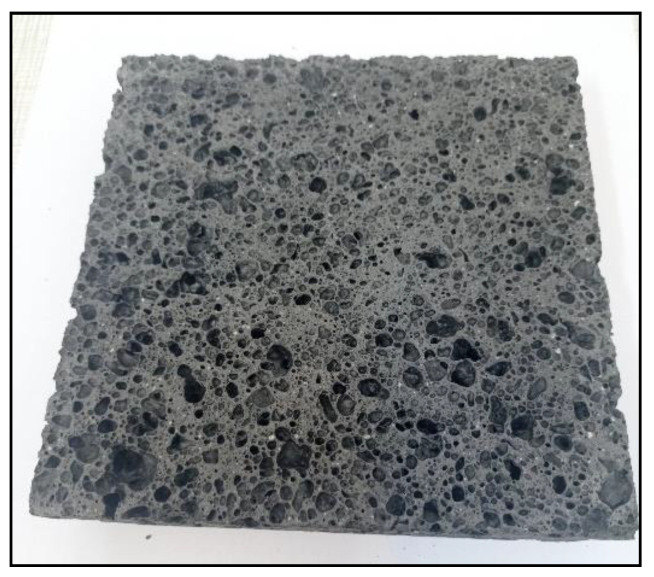
Reference sample from H_2_O_2_.

**Figure 5:**
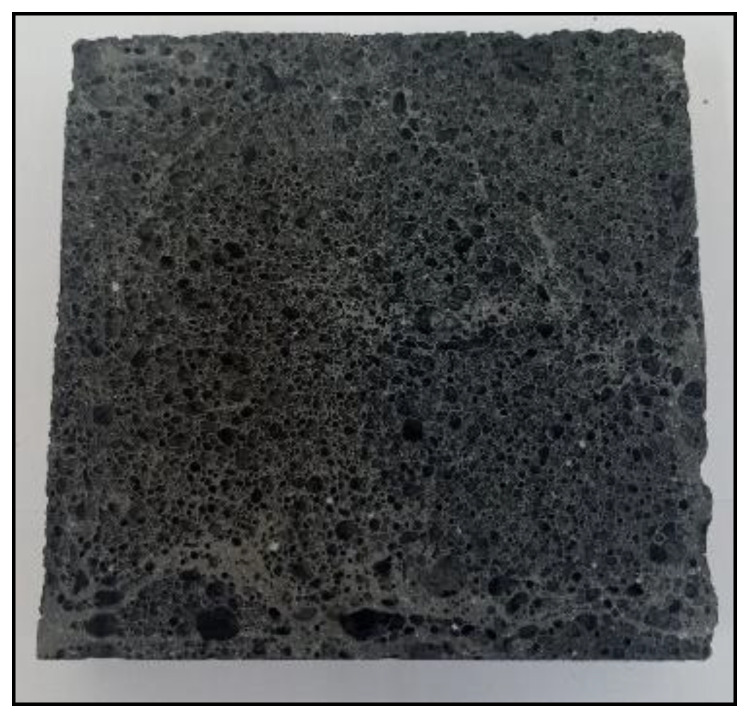
Reference sample with Al powder.

**Figure 6:**
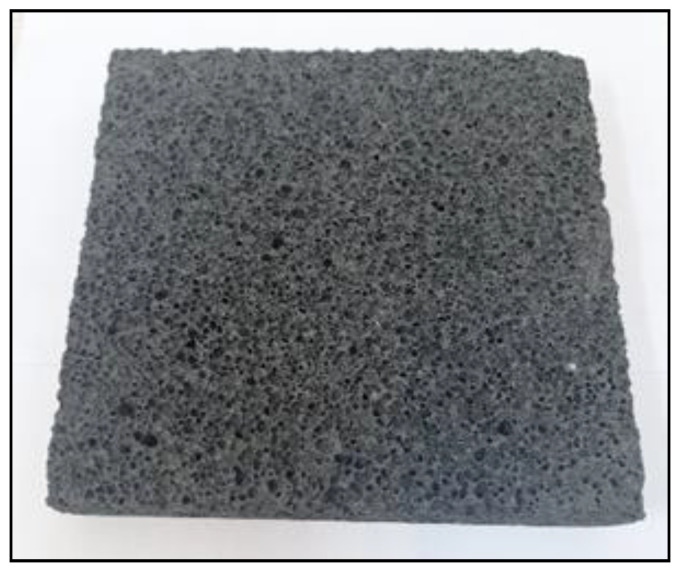
Sample from 50 g of cement.

**Figure 7:**
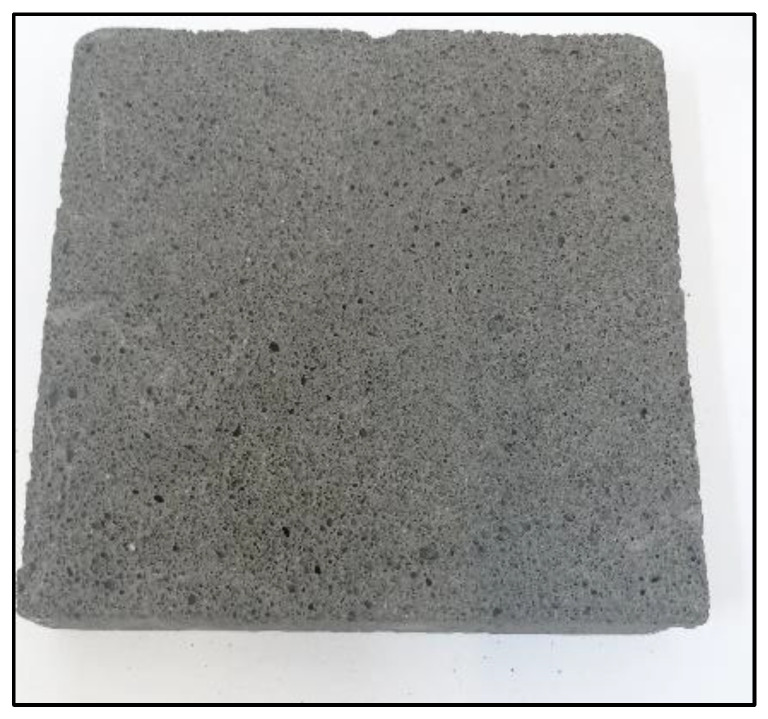
Sample from 100 g of cement.

Figure 4, Figure 5, Figure 6 and Figure 7 below show an example of the appearance of the geopolymeric foams produced. The photos show the distribution and size of the pores, as well as the color change of the samples depending on the added additives. The samples without hydraulic additives were characterized by the largest pores with irregular shapes and sizes. Their uneven distribution in the sample volume is also noticeable. The samples foamed with H_2_O_2_ than with Al powder had larger pores. The addition of cement changes the porosity into more stable, smaller, and evenly spaced pores.

**Figure 8:**
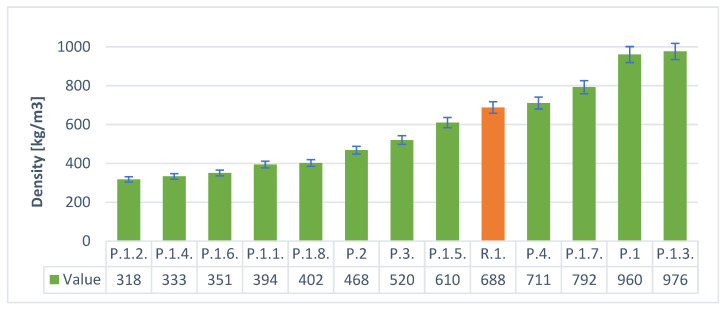
The density of samples with H_2_O_2_.

**Figure 9:**
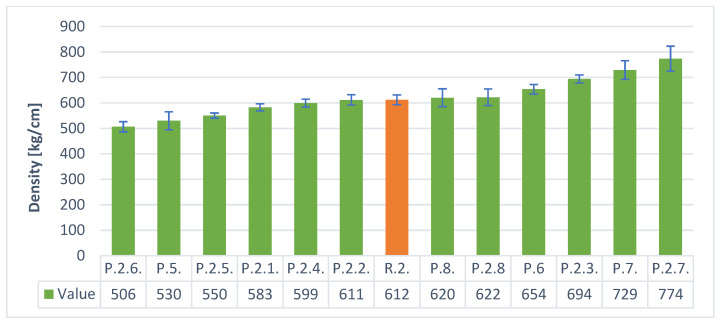
The density of samples with aluminum.

The Figure 8 and Figure 9 below show the densities of the geopolymeric foams produced with various additives. Samples P.1. and P.1.3. had the highest density, for the H_2_O_2_ frother, and the lowest P.1.2 of only 317 kg/m^3^. For the Al powder foaming variant, samples P.7 and P.2.7 had the highest density and P.2.6 had the lowest density of approximately 506 kg/m^3^. It was observed that when using the foaming agent in the form of H_2_O_2_, lower density values were obtained, which is of great importance in terms of insulation properties. It can also be observed that in the case of H_2_O_2_, despite the lower density values, there is also a large scatter of the results. It is a foaming agent very sensitive to changes in the surface tension of the geopolymeric mass; therefore, the introduced surfactants as additives had a great influence.

When using Al powder, the scatter of the results is much smaller and ranges from about 770 kg/m^3^ to 506 kg/m^3^ (the results are less dependent on the type of additives used).

**Figure 10:**
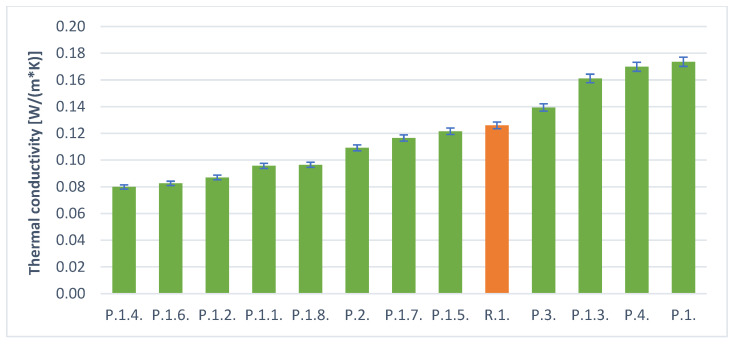
Thermal conductivity of the samples with hydrogen peroxide.

**Figure 11:**
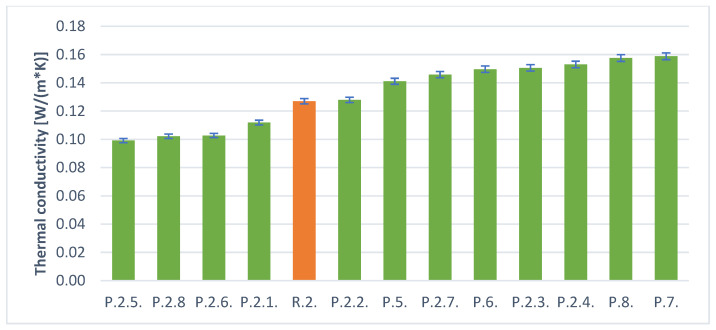
Thermal conductivity of the samples with aluminum.

Table 5 and Figure 10 and Figure 11 below present the results of the heat conduction coefficient test for foamed geopolymers with additives. The obtained values oscillate in the range from 0.17 [W/(m × K)] to 0.08 [W/(m × K)]. The results are two times worse (for the best samples) in terms of thermal insulation compared to conventional insulation materials, but it should be remembered that such materials are completely nonflammable and have much higher mechanical strength.

As a result of the tests, it was observed that better results were obtained for samples of foamed H_2_O_2_ than in the case of aluminum powder. This is also consistent with the density studies. Better insulation parameters were obtained for samples with lower density.

The addition of surfactants had a beneficial effect on reducing density and improving thermal conductivity. It should be noted that both hydraulic additives such as cement or gypsum have a positive effect on the stability of the geopolymer foams produced, but also the addition of surfactants has a positive effect. The combination of these two additives gives the best results. The samples without the addition of surfactants, but only with hydraulic stabilizers, performed worse.

The best thermal conductivity values were obtained for samples foamed with H_2_O_2_ and this was the case in several cases (P.1.2; P.1.4; P.1.6). These samples also had the lowest density of all the materials obtained. They were all obtained for the stabilizer, which was cement. It should be concluded that this is a better type of hydraulic additive than gypsum. Based on the results obtained, it can be assumed that the best solution for obtaining the best possible insulation materials from foamed geopolymers is the use of H_2_O_2_ together with cement and surfactants. However, the obtained values of thermal conductivity coefficient of 0.08 [W/m × K] are not sufficient for this material to compete with commonly used insulation materials. It is expected that the use of higher amounts of H_2_O_2_ will give better results in terms of thermal conductivity coefficient values.

Some values of thermal conductivity coefficients obtained for the tested materials in the above article are better compared to the foamed metakaolin-based geopolymer material with the addition of expanded polystyrene (0.12–0.21 [W/(m × K)]) [32]. Nezmatollahi et al. investigated the effect of adding expanded glass, perlite and ceramic microspheres embedded in a geopolymer matrix based on fly ash. The obtained values of ceipl conductivity on the above materials were 0.9 [W/(m × K)], 1.1 [W/(m × K)] and 1.1 [W/(m × K)], respectively [33].

**Figure 12:**
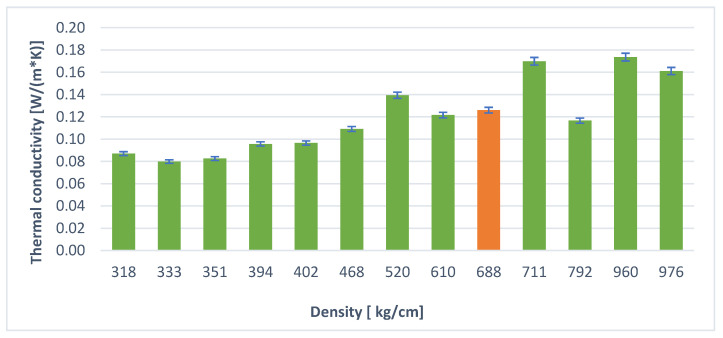
Relationship between thermal conductivity and density (samples with hydrogen peroxide).

**Figure 13:**
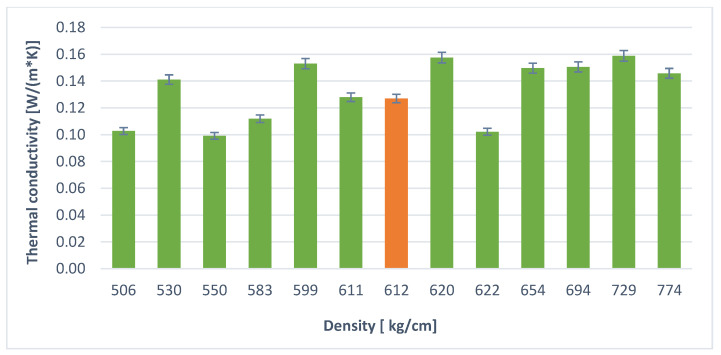
Relationship between thermal conductivity and density (samples with aluminum powder).

Investigations on fly-ash-based geopolymer foams were carried out by Feng et al. The thermal conductivity coefficient for fly ash based geopolymer foam with a foaming agent in the form of hydrogen peroxide was 0.07 [W/(m × K)] [34]. Figure 12 and Figure 13 show that in the density and thermal conductivity coefficient relationships for both the foaming agent, H_2_O_2_ and Al powder, a relationship is observed, showing a decrease in thermal conductivity with a decrease in density. Novais et al. investigated the thermal conductivity coefficients for foamed geopolymers based on fly ash and metakaolin. The values they obtained oscillated around 0.08 [W/(m × K)] for a geopolymer of density 440 kg/m^3^ and 0.22 [W/(m × K)] for a geopolymer of density 1100 kg/m^3^, respectively [21]. Wu et al. for geopolymer foams based on fly ash and metakaolin obtained results of thermal conductivity coefficient that oscillated in the range of 0.06–0.09 [W/(m × K)], at a density of 150–300 kg/m^3^ [35]. In both cases, the values of thermal conductivity were very close to each other. Research on lightweight geopolymer materials was also conducted by Wongsa et al. The values of thermal conductivity that they obtained ranged between 0.62–0.65 [W/(m × K)] for a material based on fly ash with the addition of crushed ceramic brick and 0.62–0.65 [W/(m × K)] for a geopolymer with the addition of pumice aggregate [36]. Material density and apparent porosity are correlated with the value of thermal conductivity. A higher number of pores containing air leads to a decrease in the material density and thus a decrease in the thermal conductivity value. However, some sources confirm the opposite relationship [37].

## 4. Conclusions

The results of the conducted research confirm that geopolymer foams are attractive insulating materials both for the construction industry and for other specialized applications. Although they show heat conduction coefficients about twice as high (worse) than polystyrene, what is particularly advantageous and is an advantage compared to other solutions they are more durable and completely nonflammable. A prerequisite for their mass production and wide implementation, however, is the conduct of continuous and subsequent research to optimize the processes of their products and improve the thermal conductivity, as well as the possibility of application on existing facilities. It seems that at present, the most difficulties in the production of foamed geopolymers result from the instability of these foams (their fall). The implementation of research and the influence of hydraulic additives such as Portland cement and building gypsum allowed the following conclusions to be drawn:

1. Comparing the foaming efficiency of geopolymers using hydrogen peroxide and aluminum powder as a foaming agent, it was found that better effects were obtained for H_2_O_2_—it is a better foaming agent for geopolymers than Al powder.
2. By using the hydraulic additive—stabilizer in the form of cement—lower densities and better insulation parameters were obtained than when using gypsum. Portland cement is a better stabilizer than gypsum (calcium sulfates).
3. The relationship between density and thermal conductivity is visible. As density increases, thermal conductivity increases. This relationship has also been confirmed in other previous studies by authors including: [13,15,18,38,39,40].
4. The addition of surfactants had a beneficial effect on reducing density and improving thermal conductivity. It should be noted that both hydraulic additives such as cement or gypsum have a positive effect on the stability of the geopolymer foams produced, but also the addition of surfactants has a positive effect. The combination of these two additives gives the best results. The samples without the addition of surfactants, but only with hydraulic stabilizers, performed worse.

## Figures and Tables

**Table 1:** Oxide composition of fly ash.

**Precursor**	**Oxide Composition (wt%)**							
	SiO_2_	TiO_2_	Fe_2_O_3_	Al_2_O_3_	CaO	MgO	K_2_O	Na_2_O
**Fly ash**	55.9	1.09	5.92	23.49	2.72	2.61	3.55	0.59

**Table 2:** Characterisation of stablizers: Syringaldehyde [29] and Poly(ethylene glycol) diacrylate—Average Mn 575 [30].

Name of Stablizer	Syringaldehyde	Poly(Ethylene Glycol) Diacrylate—Average Mn 575
**Brand**	Sigma-Aldrich	Aldrich
**Chemical formula**	C_9_H_10_O_4_	(C_2_H_4_O)_n_C_6_H_6_O_3_
**Molecular weight**	182.17 g/mol	-
**Appearance (Form/color)**	solid/beige	Liquid/colorless to faint yellow
**Temperature melting point**	110–113 °C	104 °C
**Temperature range boiling**	192–193 °C (in 14 mmHg)	-
**Density**	1.01 g/cm^3^	1.12 g/cm^3^ (in 25 °C)
**Application**	laboratory chemicals, production of substances	cross-linking reagent, polymerization reactions

**Table 3:** Characterization of samples with hydrogen peroxide (30 mL) as foaming additive.

Sample Name	Building Sand Mass	Fly Ash Mass	Hydraulic Additive	Stabilizer Weight	Alkaline Activator
**R.1.**	100 g	900 g	-	-	350 mL
**P.1.**	100 g	850 g	50 g gypsum	-	350 mL
**P.2.**	100 g	850 g	50 g cement	-	350 mL
**P.3.**	100 g	800 g	100 g gypsum	-	400 mL
**P.4.**	100 g	800 g	100 g cement	-	400 mL
**P.1.1.**	100 g	845 g	50 g gypsum	5 g syringaldehyde	360 mL
**P.1.2.**	100 g	845 g	50 g cement	5 g syringaldehyde	350 mL
**P.1.3.**	100 g	795 g	100 g gypsum	5 g syringaldehyde	400 mL
**P.1.4.**	100 g	795 g	100 g cement	5 g syringaldehyde	400 mL
**P.1.5.**	100 g	850 g	50 g gypsum	20 mL average Mn 575	360 mL
**P.1.6.**	100 g	850 g	50 g cement	20 mL average Mn 575	360 mL
**P.1.7.**	100 g	800 g	100 g gypsum	20 mL average Mn 575	400 mL
**P.1.8.**	100 g	800 g	100 g cement	20 mL average Mn 575	400 mL

**Table 4:** Characterization of samples with aluminum powder (1.5 g) as foaming additive.

**Sample Name**	**Building Sand Mass**	**Fly Ash Mass**	**Hydraulic Additive**	**Stabilizer Weight**	**Alkaline Activator**
**R.2.**	100 g	900 g	-	-	360 mL
**P.5.**	100 g	850 g	50 g gypsum	-	360 mL
**P.6.**	100 g	850 g	50 g cement	-	360 mL
**P.7.**	100 g	800 g	100 g gypsum	-	400 mL
**P.8.**	100 g	800 g	100 g cement	-	400 mL
**P.2.1.**	100 g	845 g	50 g gypsum	5 g syringaldehyde	365 mL
**P.2.2.**	100 g	845 g	50 g cement	5 g syringaldehyde	370 mL
**P.2.3.**	100 g	795 g	100 g gypsum	5 g syringaldehyde	400 mL
**P.2.4.**	100 g	795 g	100 g cement	5 g syringaldehyde	380 mL
**P.2.5.**	100 g	850 g	50 g gypsum	20 mL average Mn 575	360 mL
**P.2.6.**	100 g	850 g	50 g cement	20 mL average Mn 575	360 mL
**P.2.7.**	100 g	800 g	100 g gypsum	20 mL average Mn 575	380 mL
**P.2.8.**	100 g	800 g	100 g cement	20 mL average Mn 575	380 mL

**Table 5:** Coefficient of thermal conductivity for samples with H_2_O_2_ and aluminum.

Foaming Agent	Sample Determination	Results [W/(m × K)]	Foaming Agent	Sample Determination	Results [W/(m × K)]
H_2_O_2_ (30 mL)	R.1.	0.12596	Al powder (1.5 g)	R.2.	0.12698
	P.1.	0.17359		P.5.	0.14107
	P.2.	0.10913		P.6.	0.14961
	P.3.	0.13936		P.7.	0.15875
	P.4.	0.16986		P.8.	0.15750
	P.1.1.	0.09564		P.2.1.	0.11186
	P.1.2.	0.08696		P.2.2.	0.12789
	P.1.3.	0.16108		P.2.3.	0.15054
	P.1.4.	0.07985		P.2.4.	0.15299
	P.1.5.	0.12154		P.2.5.	0.09914
	P.1.6.	0.08251		P.2.6.	0.10271
	P.1.7.	0.11655		P.2.7.	0.14576
	P.1.8.	0.09647		P.2.8.	0.10214
